# Depression among patients with ankylosing spondylitis in southern Tunisia: Prevalence and associated factors

**DOI:** 10.1192/j.eurpsy.2024.1096

**Published:** 2024-08-27

**Authors:** A. Feki, I. SELLAMI, M. Baklouti, N. Ketata, Z. Gassara, S. Ben jemaa, M. Ezzeddine, M. H. Kallel, H. Fourati, R. Akrout, S. Baklouti, Y. Mejdoub

**Affiliations:** ^1^Rheumatology; ^2^occupational medicine; ^3^Community Medicine and Epidemiology Department, Hedi chaker hospital, Sfax, Tunisia

## Abstract

**Introduction:**

Ankylosing spondylitis (AS) is one of the most common inflammatory rheumatisms. It is a chronic, sometimes disabling and it could cause both physical and psychological problems among patients, including depression.

**Objectives:**

With this in mind, the objective of our work was to study the prevalence of depression among patients with AS and to determine its associated factors.

**Methods:**

This was a retrospective descriptive and analytical study, carried out in 2021 over a period of 5 years in southern Tunisia on patients with a confirmed diagnosis of AS established in accordance with the ASAS diagnostic criteria (Assessment of Spondyloarthritis International Society) or the modified New York criteria for AS. Depression was assessed using the *Hospital anxiety and Depression (HAD) score.* A HAD score>10 means certain depression.

**Results:**

A total of 62 patients were included in our study. The median age was 39 years with an interquartile range (IQR) = [32-50 years]. There were 35 men (56.5%). Inflammatory back pain was noted among 51 patients (82.3%). Extraarticular manifestations were noted among 14 cases (22.6%) and were mainly ocular (11 cases; 78.4%). The diagnosis was confirmed by ASAS criteria in 55 cases (88.7%). AS was treated symptomatically in 58 cases (93.5%), specifically by basic treatment among 17 patients (27.4%) and by additional physical rehabilitation among 15 patients (24.2%). Depression was certain among 30 patients, giving a global prevalence of 48.4%. The factors statistically associated with this disease among patients with AS were having a low level of education (illiterate or primary) (Odds Ratio (OR) = 2.87; p = 0.044), being clinically suffering from severe fatigue (OR= 7.14; p<0.001), have a poor quality of life [Ankylosing spondylitis quality of life questionnaire (Asqol) Score ≥13] (OR=4.52; p=0.007) and have certain anxiety (HAD>10) (OR=19; p<0.001).

**Conclusions:**

In addition to its clinical impact on patients, the psychological impact of AS was considerable in terms of depression. The factors associated with it were individual, clinical, and psychological. Thus, psychological support must be coupled with AS medical management in order to prevent psychological disorders among patients, particularly depression.

**Disclosure of Interest:**

None Declared

